# Sub-Acute Oral Toxicity of a Novel Derivative of Agomelatine in Rats in a Sex-Dependent Manner

**DOI:** 10.3389/fphar.2019.00242

**Published:** 2019-03-19

**Authors:** Qiushi Yang, Xuelin Zhou, Jingyi Li, Yi Ma, Li Lu, Jie Xiong, Pingxiang Xu, Yuhang Li, Yi Chen, Wei Gu, Ming Xue, Zengliang Jin, Xiaorong Li

**Affiliations:** ^1^Department of Pharmacology, School of Basic Medical Sciences, Capital Medical University, Beijing, China; ^2^Beijing Friendship Hospital, Capital Medical University, Beijing, China; ^3^Experimental Center for Basic Medical Teaching, School of Basic Medical Sciences, Capital Medical University, Beijing, China; ^4^Beijing Guangwei Pharmaceutical Technology Co., Ltd., Beijing, China

**Keywords:** antidepressant, agomelatine, derivative, toxicity, GW117

## Abstract

Agomelatine (AGO) is a new type of antidepressant with demonstrated antidepressant effects and a unique modulating circadian rhythm action. However, AGO has hepatotoxicity, which limits its clinical application. In order to develop new drugs that cause less liver injury than AGO, a series of derivatives were synthesized; compound GW117 was screened from derivatives due to its high receptor affinity. This study will investigate its sub-acute oral toxicity profile in rats in a sex-dependent manner. GW117 and AGO was administrated by gavage (200, 400, or 800 mg/kg/day) for 28 days. Hematological, biochemical tests, organ weights, histopathological examinations were carried out, the results showed that AGO and GW117 had adverse effects on platelet, liver and kidney, and had sex-differences in some indicators. Hematological tests showed that AGO and GW117 reduced the platelet count in male animals but had no effect in females. AGO increased plasma alanine aminotransferase (ALT) and total bilirubin in male animals, and GW117 had no effect on these two indicators. For females, AGO moderately elevated ALT, alkaline phosphatase (ALP), and total bilirubin, while GW117 only elevated ALP slightly. Two drugs could increase liver weight and coefficient, and cause liver pathological injury, including hepatic sinusoidal dilatation, hepatocyte fatty deposition and dotted cell necrosis in two genders. AGO caused mild to moderate hepatocyte and hepatobiliary injury in both genders, while only a mild hepatobiliary injury was caused by GW117 in females. Renal function tests showed that both drugs can increase blood urea nitrogen levels in males, while AGO, but not GW117, can slightly increase blood creatinine and urea nitrogen in females. The kidney weight and coefficient could be significantly increased by two drugs in males, and by AGO medium and GW117 high and low doses in females. The kidney pathological damage was mainly characterized by tubule dilatation, a thinning of the renal cortex. Kidney damage caused by GW117 was less than that of AGO, and there was no sex-difference. In summary, GW117 can cause mild liver and kidney damage in both genders, as well as mild platelets reduction in males, while degree of damage is less severe than AGO. Therefore, as an excellent derivative, GW117 deserves further development as an antidepressant.

## Introduction

**FIGURE 1:**
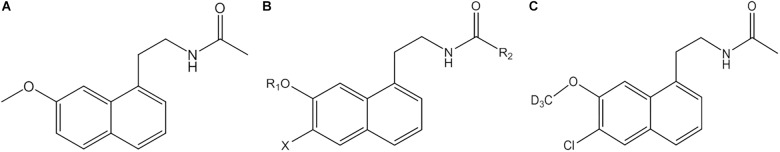
Structure of AGO **(A)**, structure of the series derivatives of AGO **(B)**, and GW117 **(C)**. X are H or halogen atoms; R_1_ are CH_3_ or CD_3_; R_2_ are CH_3_, CD_3_, or C_2_H_5_.

**FIGURE 2:**
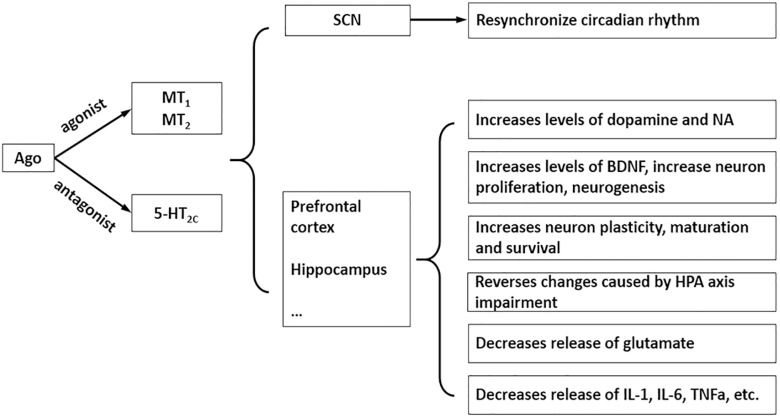
Chief mechanisms and pharmacological properties of AGO. SCN: suprachiasmatic nucleus.

Agomelatine (AGO) (N[2-(7-methoxy-1-naphthyl)ethyl] acetamide) (Figure 1A) is a new type of antidepressant with dual mechanisms of action: melatonin MT_1_ and MT_2_ receptor agonist and 5-HT_2C_ receptor antagonist (Millan et al., 2003). It has good antidepressant, anxiolytic and procognitive effects, although the precise mechanism needs further research, accumulating evidence supports the notion that its psychotropic effects are due to multiple pharmacological properties that rely on a synergistic action at both melatonin and 5-HT_2C_ receptors (Figure 2; Rainer et al., 2012; Milanese et al., 2013; Molteni et al., 2013; Guardiola-Lemaitre et al., 2014; Boulle et al., 2016; Martinotti et al., 2016; De Berardis et al., 2017). Currently the most commonly used antidepressant drugs are selective serotonin reuptake inhibitors (SSRIs), while other types include tricyclic antidepressants (TCAs), noradrenaline reuptake inhibitor (NARI), serotonin and noradrenaline reuptake inhibitor (SNRI), noradrenergic and specific serotonergic antidepressants (NaSSA), etc. So far, the effective rate of antidepressants is 60∼80%, but has a low cure rate (about 30%); the common on set time of action is 2∼4 weeks, and it is easy to develop drug dependence and or to experience sexual function issues (Qiang and Aixia, 2016). AGO provides a new idea for antidepressant treatment, which was approved by the European Medicines Agency for the treatment of major depression in adults in February 2009 (Carney and Shelton, 2011). AGO agonizes the MT_1_ and MT_2_ receptors to synchronize the biorhythm, increases the sleeping time, and reduces the number of awakenings to relieve depression-associated sleep disorders. With the action of antagonism of 5-HT_2C_ receptors, it can inhibit the binding and uptake of the 5-HT by 5-HT_2C_ receptor and can increases the concentration of norepinephrine and dopamine in the prefrontal lobe (Millan et al., 2003), and also significantly reduces symptoms in patients with depression (Medvedev, 2017). Not only can AGO be used alone, but it can also be combined with other antidepressants to further improve antidepressant effects.

In general, its safety and tolerability profile appears to be favorable or similar than other antidepressants, particularly regarding a lack of withdrawal syndrome (Montgomery et al., 2004) and low sexual dysfunction, which makes it a useful alternative for treating depressive disorder in sexually active men (Bissell, 2004; Zormann et al., 2016). However, AGO and many other antidepressants may induce hepatotoxicity, even at a therapeutic dose, among antidepressants initially proposed as first-line therapy for major depressive disorders (Voican et al., 2014), such as fluoxetine, sertraline (SSRI) (Almansour et al., 2018; Elgebaly et al., 2018), imipramine (TCA) (He et al., 2015), venlafaxine, and duloxetine (SNRI) (Xue et al., 2011; Park and Ishino, 2013). Epidemiology and the pathophysiology of AGO-related hepatotoxicity is currently poorly understood, unpredictable and usually occurs during the first month of treatment. After long-term administration, the most common symptoms are nausea, fatigue, loss of appetite, and abdominal pain (Friedrich et al., 2016), and 0–0.6 and 3–4.5% of patients treated with 25 or 50 mg showed elevated transaminases, respectively. Incidence rates of serious liver injury caused by AGO manifests as hepatitis, jaundice, and hepatic failure was much higher than SSRIs (Freiesleben and Furczyk, 2015; de Gage et al., 2018). The underlying mechanism appears to be idiosyncratic (Gahr et al., 2014). In a recent post-authorization opinion of the European Medicines Agency, the hepatotoxic reactions related to AGO were declared as an important identified risk, and recommended that liver function tests should be performed in all patients before and during the treatment regularly (Zormann et al., 2016). It is suggested that AGO should be discontinued if the increase of transaminase is more than three times the upper limit of normal (ULN) value (Gahr et al., 2014; European Medicines Agency, 2017). Careful evaluation of the benefit–risk must be made before AGO is administered in patients with pre-existing liver diseases including fatty liver, obesity, diabetes mellitus, substantial alcohol intake and concomitant use of drugs with hepatotoxic potential (Perlemuter et al., 2016). To date, AGO is the only antidepressant requiring liver monitoring during treatment. Therefore, we attempted to develop an effective alternative with lower hepatic toxicity. On the basis of the chemical structure of AGO, a series of compounds have been synthesized (Figure 1B), as indicated in the international patent (No. PCT/CN2018/095325). GW117 (Figure 1C) was screened by receptor binding experiments with highest binding affinity found among the derivatives. GW-117 dose-dependently inhibited the binding of [^3^H]-mesulergine to 5-HT_2C_ and [^3^H]-melatonin to MT_1_ and hMT_2_, with respective inhibitory rates of 98, 83, and 78% at 100 nM (These data will be published in another article). In the current study, to further evaluate the general toxicity and liver damage of GW117, a 28-day toxicity study was carried out to understand the toxicological properties in rats. Since previous studies suggested some kind of gender differences, the toxic effects on two genders will be observed separately.

## Materials and Methods

### Reagents

Agomelatine and GW117 were supplied by Beijing Guangwei Pharmaceutical Technology Co., Ltd. The suspension of the corresponding concentration was prepared using 0.5% sodium carboxymethylcellulose.

### Animals

84 Sprague-Dawley rats (7-week-old; male and female; weight 160 ± 25 g) were provided by the Experimental Animal Department of Capital Medical University. Animals were caged by sex (3 per cage) in the Specific Pathogen Free animal laboratory (room temperature, 23 ± 3°C; humidity, 55 ± 15%) with free access to food and water. The experiments were carried out in accordance with the current guidelines for the care of laboratory animals and the ethical guidelines for investigations of experiments in conscious animals. In addition, the protocols employed were approved by the Animal Care and Use Committee of Capital Medical University (Approved number AEEI-2016-159).

### Animal Grouping and Drug Administration

Male and female rats (*n* = 84) were randomly divided into seven groups, including controls treated with vehicle (0.5% CMC), AGO 200, 400, and 800 mg/kg and GW117 200, 400, and 800 mg/kg. Each group was given the corresponding drug suspension by gavage at 9 am every day for 28 days, the volume was 1 ml/100 g, and the vehicle control group was given an equal volume of 0.5% CMC suspension. The dose of AGO were approximately 80, 160, and 320 times to its clinical dose, and GW117 doses were set the same as AGO.

### Sampling and Index Detection

The animals feed intake and fecal traits, were recorded daily. Body weight was recorded twice a week. After the last administration, the animals were fasted for 12 h and anesthetized with 7% chloral hydrate. Blood samples of all animals were collected in different tubes from the abdominal aorta. Full blood samples were collected into a 2 ml EDTA-treated tube for hematological analysis with sysmex 5000 clinical hematology analyzers (Japan). A 5 ml blood sample was collected into heparin-treated tubes and centrifuged at 3500 rpm for 10 min to obtain the plasma for biochemical assays using the Beckman Coulter AU5821 automatic biochemical analyzer (United States). Brain, heart, liver, spleen, lung, kidney, testis, and uterus were collected and weighed to calculate organ weight coefficient. Part of the tissues from three rats of each group were taken and fixed immediately in 4% paraformaldehyde, dehydrated in graded alcohol (70, 90, 95, and 100%) and embedded in paraffin. The embedded tissues were then cut into 5 μm thick sections and stained with hematoxylin-eosin for histopathological analysis. The organ weight coefficient was calculated as: [organ weight (g) ÷ body weight (g)] × 100. The histopathology slides were viewed using software CaseViewer, and the percentage of dilated areas of hepatic sinuses was calculated as: [The sum of the areas of the bile duct dilated areas ÷ total areas of slide] × 100% by ImageJ.

### Statistical Analysis

Data are expressed as mean ± SD. Statistical analysis was performed using One-Way Analysis of Variance (ANOVA) followed by the Least Significance Difference (LSD) *post hoc* test by SPSS 20. The body weight analysis was performed using General Linear Model/Repeated Measures, and a *p*-value lower than 0.05 was established as a statistically significant difference. Pearson correlation analysis was applied to assess the correlation between the liver coefficient and drug doses administered.

## Results

### Effects of Ago and GW117 on the General Signs and Body Weight

**FIGURE 3:**
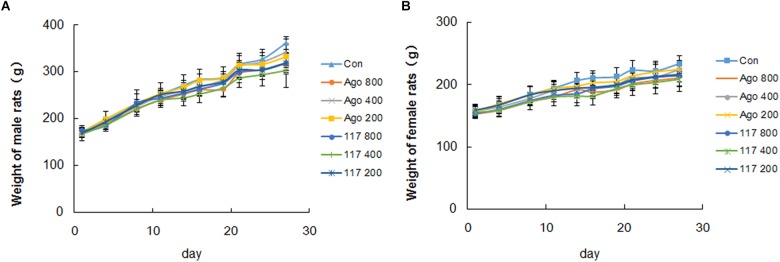
Effect of AGO and GW117 on the body weight of male **(A)** and female **(B)** rats (*n* = 6).

There was no obvious abnormality in animal appearance, behavior, food intake or fecal traits during the whole experimental period. Animals moved freely, breathed evenly and showed lustrous and smooth fur, and responded with sensitivity to external stimulus, with normal characteristics of defecation and urination. The body weight of all genders in each group increased evenly during the experiment (Figure 3A,B). The body weight of male and female animals in the vehicle control group increased gradually from 170.3 ± 10.6 to 361.6 ± 13.1 g and from 157.3 ± 10.9 to 233.8 ± 12.5 g, respectively. The weight growth trend of animals in other groups were similar to that in the control group with no significant difference. There was no statistical difference in the effect of GW117 and AGO on the trend of changes in body weight during the experiment.

### Effects of Ago and GW117 on the Hematological Parameters

As shown in Table 1, for male animals, all groups of GW117 and AGO had no negative effect on the levels of erythrocyte count, erythrocyte volume, hemoglobin content related to red blood cells, and had no effect on WBC, lymphocyte ratitio, and neutrophil ratio. However, GW117 and AGO decreased the count of platelet and platelet hematocrit statistically in male animals when compared to the control group (*p* < 0.01), but did not influence the platelet volume. For female animals, only the mean hemoglobin concentration was decreased statistically by AGO at 800 mg/kg. Overall, GW117 and AGO have a more obvious decreasing effect on platelets in male animals, not in female animals.

**Table 1:** Effects of AGO and GW117 on hematological parameters in rats (*n* = 6).

Group	Dose (mg/kg)	Gender	RBC (10^12^/L)	WBC (10^9^/L)	HGB (g/L)	PLT (10^9^/L)	HCT (%)	MCV (fL)	MCH (pg)	MCHC (g/L)	RDW (%)	LY (%)	MO (%)	GR (%)	PCT (%)	MPV (fL)	PDW (%)
Vehicle	0.5% CMC	Male	7.01 ± 0.41	10.47 ± 2.30	151.17 ± 8.61	914.67 ± 87.23	43.73 ± 2.65	62.37 ± 1.11	21.57 ± 0.51	345.67 ± 7.47	14.52 ± 0.71	84.08 ± 4.80	10.48 ± 1.86	5.44 ± 4.26	0.38 ± 0.05	4.18 ± 0.31	16.53 ± 1.24
AGO	800		7.47 ± 0.38	7.20 ± 3.05	155.50 ± 9.94	742.50 ± 104.10^∗^	45.3 ± 2.975	60.63 ± 1.61	20.80 ± 0.55	343.17 ± 6.80	15.38 ± 0.45	81.98 ± 3.48	12.90 ± 2.87	5.13 ± 3.16	0.27 ± 0.03^∗^	3.67 ± 0.16	15.53 ± 0.23
	400		7.40 ± 0.31	8.44 ± 1.94	151.80 ± 7.26	744.40 ± 82.63^∗^	44.30 ± 1.92	59.94 ± 3.05	20.52 ± 0.74	342.40 ± 7.40	14.84 ± 0.63	82.53 ± 1.29	9.43 ± 3.44	8.03 ± 3.18	0.29 ± 0.04^∗^	3.96 ± 0.39	15.74 ± 0.47
	200		7.20 ± 0.33	8.38 ± 3.77	150.40 ± 6.19	770.20 ± 65.62^∗^	43.38 ± 1.75	60.30 ± 1.01	20.90 ± 0.54	347.00 ± 8.16	14.04 ± 0.31	76.90 ± 11.95	12.03 ± 3.95	11.07 ± 7.80	0.28 ± 0.05^∗^	3.68 ± 0.56	14.98 ± 0.68^∗^
GW117	800		7.09 ± 0.28	12.00 ± 2.35	149.00 ± 7.43	787.00 ± 90.50^∗^	43.80 ± 2.06	61.83 ± 2.07	21.02 ± 0.58	340.00 ± 9.47	15.52 ± 0.87	75.86 ± 5.89	15.02 ± 2.09	9.12 ± 4.21	0.27 ± 0.04^∗^	3.48 ± 0.32^∗^	15.73 ± 0.66
	400		7.33 ± 0.29	11.95 ± 3.07	152.00 ± 4.94	790.00 ± 106.07^∗^	43.73 ± 1.63	59.67 ± 0.64	20.75 ± 0.17	347.67 ± 4.50	14.37 ± 0.38	80.57 ± 3.89	13.30 ± 1.67	6.13 ± 4.99	0.30 ± 0.07^∗^	3.87 ± 0.34	15.38 ± 0.68
	200		7.43 ± 0.26	11.05 ± 3.92	153.50 ± 5.54	764.67 ± 60.07^∗^	44.40 ± 1.47	59.80 ± 1.80	20.67 ± 0.83	345.83 ± 4.36	14.40 ± 0.80	76.30 ± 17.17	17.10 ± 6.70	6.6 ± 10.60	0.30 ± 0.05^∗^	4.00 ± 0.44	16.50 ± 0.32
Vehicle	0.5% CMC	Female	6.80 ± 0.46	7.03 ± 1.43	147.83 ± 6.05	691.50 ± 57.70	41.42 ± 1.68	61.05 ± 2.74	21.80 ± 1.22	357.17 ± 8.26	14.17 ± 0.67	81.10 ± 6.69	10.74 ± 1.35	8.16 ± 5.68	0.24 ± 0.03	3.55 ± 0.53	16.02 ± 0.73
AGO	800		6.70 ± 1.17	5.62 ± 3.00	140.83 ± 25.17	787.33 ± 224.41	41.20 ± 7.25	61.47 ± 1.44	20.98 ± 0.44	341.83 ± 2.14^∗∗^	14.17 ± 0.52	78.30 ± 5.63	11.86 ± 2.27	9.84 ± 4.33	0.27 ± 0.07	3.50 ± 0.33	15.28 ± 0.48
	400		7.24 ± 0.46	7.70 ± 1.46	152.83 ± 6.24	793.33 ± 85.87	43.40 ± 1.38	60.00 ± 2.36	21.12 ± 0.64	352.17 ± 8.80	14.18 ± 0.80	78.13 ± 7.81	12.87 ± 1.96	9.00 ± 6.55	0.26 ± 0.06	3.28 ± 0.38	15.22 ± 0.48
	200		7.26 ± 0.31	6.38 ± 1.49	155.33 ± 6.06	779.00 ± 83.24	44.18 ± 2.05	60.90 ± 1.17	21.43 ± 0.51	351.67 ± 4.32	14.48 ± 0.79	87.33 ± 3.03	10.03 ± 3.35	2.63 ± 1.56	0.26 ± 0.05	3.37 ± 0.52	15.38 ± 0.78
GW117	800		7.01 ± 0.24	5.56 ± 1.67	144.20 ± 3.11	773.40 ± 69.27	41.20 ± 1.39	58.78 ± 2.21	20.56 ± 0.67	350.40 ± 5.60	14.54 ± 0.59	87.05 ± 3.75	11.15 ± 2.76	1.80 ± 0.99	0.25 ± 0.05	3.24 ± 0.41	15.16 ± 0.52
	400		7.09 ± 0.42	8.60 ± 2.51	148.00 ± 6.54	856.33 ± 93.08	42.48 ± 1.84	59.97 ± 1.48	20.90 ± 0.62	348.33 ± 7.03	14.05 ± 0.62	70.80 ± 14.63	13.80 ± 2.33	15.40 ± 13.53	0.29 ± 0.05	3.40 ± 0.27	14.92 ± 0.45
	200		6.93 ± 0.33	9.50 ± 3.81	152.00 ± 8.46	798.80 ± 97.43	42.82 ± 1.84	61.84 ± 1.92	21.94 ± 0.33	355.00 ± 10.32	13.86 ± 0.69	76.40 ± 15.47	14.08 ± 4.60	9.53 ± 11.01	0.30 ± 0.02^∗^	3.84 ± 0.65	15.54 ± 1.07


*Vs. 0.5% CMC group,^∗^*p* < 0.05, ^∗∗^*p* < 0.01. RBC, red blood cell count; WBC, white blood cell count; HGB, hemoglobin content; PLT, platelet count; HCT, hematocrit; MCV, erythrocyte mean corpuscular volume; MCH, mean cell hemoglobin; MCHC, mean hemoglobin concentration; RDW, red blood cell distribution width; LY%, lymphocyte ratio; MO%, monocyte ratio; GR%, neutrophil ratio; PCT, platelet hematocrit; MPV, mean platelet volume; PDW, platelet distribution width.*

### Effects of Ago and GW117 on the Serum Biochemical Parameters

In Table 2, it can be seen that AGO at 800 mg/kg in male rats increased alanine aminotransferase (ALT) and total bilirubin by 1.76 (*p* < 0.01) and 3.45 (*p* < 0.01) times than that of the control group, respectively. AGO at 400 mg/kg increased ALT and total bilirubin by 1.44 (*p* < 0.05) and 1.67 times (*p* < 0.05). AGO 200 mg/kg and three doses of GW117 had no effect on ALT and total bilirubin serum levels. Both drugs had no effect on the level of aspartate aminotransferase in male animals. For female animals, the high, medium doses of AGO could significantly increase the serum ALT and total bilirubin level (*p* < 0.01), but less than three times. GW117 did not affect these two indicators. Two drugs did not affect plasma aspartate aminotransferase levels in female animals. AGO at a high and low dose and GW117 at a low dose could increase the levels of ALP (*p* < 0.05). Ago increased ALT, ALP and Total Bilirubin simultaneously, while GW117 only increased ALP in female rats.

**Table 2:** Effects of AGO and GW117 on blood biochemical parameters in rats (*n* = 6).

Group	Dose (mg/kg)	Gender	ALT (IU/L)	AST (IU/L)	ALP (IU/L)	Total bilirubin (IU/L)	Total protein (g/L)	Albumin (g/L)	Prealbumin (mg/L)	Uric acid (μ mol/L)	Creatinine (μ mol/L)	Urea nitrogen (mmol/L)	CHO (mmol/L)	TG (mmol/L)	HDL (mmol/L)	LDL (mmol/L)
Vehicle	0.5% CMC	Male	40.40 ± 6.38	89.23 ± 10.74	202.0 ± 41.6	1.28 ± 0.41	56.32 ± 2.02	40.38 ± 1.29	13.27 ± 2.34	60.50 ± 21.49	48.05 ± 3.09	5.04 ± 0.47	1.52 ± 0.18	0.29 ± 0.08	0.98 ± 0.10	0.59 ± 0.13
AGO	800		71.50 ± 9.76^∗∗^	96.60 ± 16.84	245.0 ± 39.4	4.42 ± 1.07^∗∗^	57.73 ± 3.97	38.83 ± 2.39	14.40 ± 1.57	53.00 ± 25.29	53.44 ± 7.40	7.40 ± 1.30^∗∗^	1.42 ± 0.33	0.33 ± 0.11	1.01 ± 0.22	0.45 ± 0.18
	400		58.38 ± 10.76^∗^	98.48 ± 16.70	229.6 ± 16.7	2.14 ± 0.72^∗^	56.82 ± 1.42	41.00 ± 2.42	13.24 ± 2.33	48.00 ± 7.21	46.12 ± 5.27	6.69 ± 1.30^∗^	1.21 ± 0.30	0.54 ± 0.16^∗∗^	0.93 ± 0.14	0.28 ± 0.15
	200		46.64 ± 3.32	107.50 ± 30.57	189.0 ± 68.5	1.06 ± 0.28	54.06 ± 2.38	38.20 ± 2.60	12.88 ± 1.83	43.40 ± 7.77	43.70 ± 4.07	5.39 ± 0.70	1.38 ± 0.08	0.36 ± 0.08	0.90 ± 0.08	0.54 ± 0.08
GW117	800		51.50 ± 12.70	84.43 ± 7.13	198.3 ± 37.1	1.19 ± 0.52	57.28 ± 2.10	40.68 ± 1.60	13.62 ± 1.09	52.67 ± 12.85	40.98 ± 4.85	5.42 ± 0.47	1.71 ± 0.38	0.34 ± 0.09	1.15 ± 0.22	0.50 ± 0.14
	400		35.17 ± 16.91	65.93 ± 31.25	205.0 ± 60.6	1.30 ± 0.65	57.30 ± 2.28	38.08 ± 3.93	15.67 ± 1.60	58.83 ± 13.14	47.37 ± 6.40	6.76 ± 1.33^∗^	1.39 ± 0.38	0.33 ± 0.11	0.89 ± 0.17	0.51 ± 0.19
	200		41.75 ± 8.73	87.52 ± 17.72	176.0 ± 6.4	1.11 ± 0.38	54.82 ± 1.43	39.03 ± 0.77	17.88 ± 2.21^∗∗##^	49.50 ± 7.09	44.22 ± 4.62	5.59 ± 1.26	1.40 ± 0.31	0.33 ± 0.06	0.81 ± 0.14	0.62 ± 0.15
Vehicle	0.5% CMC	Female	31.48 ± 2.40	78.68 ± 4.42	75.4 ± 4.5	1.47 ± 0.51	58.20 ± 3.59	42.75 ± 2.10	19.72 ± 2.21	48.00 ± 8.79	45.82 ± 5.30	6.66 ± 0.47	1.66 ± 0.25	0.29 ± 0.09	1.33 ± 0.20	0.42 ± 0.10
AGO	800		67.17 ± 7.54^∗∗^	110.75 ± 53.00	116.7 ± 53.0^∗∗^	6.56 ± 1.29^∗∗^	63.50 ± 3.46^∗^	46.70 ± 2.51^∗∗^	24.53 ± 3.69^∗∗^	44.00 ± 13.70	59.07 ± 6.60^∗∗^	10.46 ± 1.25^∗∗^	2.34 ± 0.73^∗^	0.48 ± 0.20	1.71 ± 0.46^∗^	0.49 ± 0.16
	400		55.62 ± 16.71^∗∗^	81.2 ± 13.92	69.5 ± 13.5	3.48 ± 0.73^∗∗^	56.97 ± 1.69	42.53 ± 2.44	26.08 ± 2.15^∗∗^	49.67 ± 14.83	52.88 ± 6.57^∗^	7.06 ± 1.04	2.06 ± 0.23	0.73 ± 0.52^∗^	1.52 ± 0.25	0.46 ± 0.16
	200		42.90 ± 7.55	105.56 ± 37.30	105.9 ± 37.3^∗^	1.43 ± 0.64	57.05 ± 1.69	41.58 ± 1.57	29.15 ± 2.89^∗∗^	38.17 ± 8.80	49.27 ± 2.94	7.53 ± 1.02	1.67 ± 0.50	0.37 ± 0.13	1.27 ± 0.37	0.50 ± 0.15
GW117	800		38.83 ± 15.50	79.43 ± 29.58	80.2 ± 29.0	1.25 ± 0.09	60.78 ± 2.40	45.32 ± 1.78	32.16 ± 0.83^∗∗##^	50.00 ± 13.13	47.08 ± 5.89	8.93 ± 1.81^∗#^	2.22 ± 0.46^∗^	0.43 ± 0.19	1.74 ± 0.26^∗^	0.35 ± 0.07
	400		42.70 ± 12.28	73.92 ± 17.77	88.8 ± 17.0	0.99 ± 0.46	58.18 ± 3.86	42.17 ± 3.13	33.75 ± 1.72^∗∗##^	45.67 ± 6.44	46.53 ± 2.80	6.28 ± 1.11	2.00 ± 0.32	0.81 ± 0.45^∗∗^	1.52 ± 0.19	0.43 ± 0.09
	200		41.78 ± 14.47	57.46 ± 26.55	114.8 ± 26.6^∗∗^	0.98 ± 0.39	56.18 ± 2.65	40.66 ± 1.79	33.68 ± 1.64^∗∗##^	33.60 ± 7.13	42.56 ± 6.46	6.98 ± 0.95	1.92 ± 0.18	0.65 ± 0.28	1.50 ± 0.09	0.43 ± 0.09


*Vs. 0.5% CMC group,^∗^*p* < 0.05, ^∗∗^*p* < 0.01 vs. the same dose of AGO group, ^#^*p* < 0.05, ^##^*p* < 0.05. ALT, alanine aminotransferase; AST, aspartate aminotransferase; ALP, alkaline phosphatase; CHO, cholesterol; TG, triglyceride; HDL, high density lipoprotein; LDL, low density lipoprotein.*

For total protein and albumin as indices of liver protein synthesis function, AGO had little influence, only AGO at 800 mg/kg could increase these two indicator levels in the females, while GW117 did not affect the levels in any of the genders. For prealbumin, three doses of AGO and GW117 could significantly increase its level in female animals, and GW117 showed a stronger effect than AGO. Elevated prealbumin may indicate an increased metabolism in the liver, with no clinical significance associated with liver injury.

These two drugs had very little negative effects on plasma lipid levels in all genders (Table 2). There was a mild increase of triglycerides (TG) in the AGO 400 mg/kg group in males. For female animals, there was a mild increase of cholesterol (CHO) in the AGO 800 mg/kg and GW117 400 mg/kg groups and an increase of the TG level in the AGO 200 mg/kg and GW117 200 mg/kg groups.

With respect to three plasma markers representing renal function, both drugs had no effect on plasma uric acid levels in all genders (Table 2). For male animals, AGO at 800 and 400 mg/kg, GW117 at 400 mg/kg increased blood urea nitrogen levels. Both had no effects on serum creatinine levels. For female animals, AGO at 800 and 400 mg/kg significantly increased creatinine levels. AGO at 800 mg/kg and GW117 at 800 mg/kg increased blood urea nitrogen levels.

### Effects of Ago and GW117 on the Organ Weight and Coefficient

**FIGURE 4:**
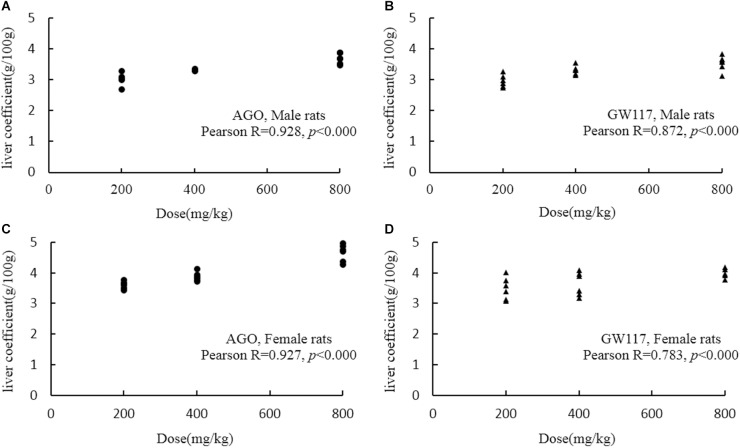
Scatter graph and Pearson correlation coefficient between liver coefficient and doses of AGO or GW117 (*n* = 6). **(A)** Correlation between liver coefficient and doses of AGO in male rats; **(B)** correlation between liver coefficient and doses of GW117 in male rats; **(C)** correlation between liver coefficient and doses of AGO in female rats; **(D)** correlation between liver coefficient and doses of GW117 in female rats.

In Table 3, GW117 and AGO had very little effect on the weight and organ coefficient of the brain, heart, spleen, lungs and testes/ovary. The main organs affected by the two drugs are the liver and kidneys. Three doses of AGO and GW117 could significantly increase the liver coefficient in male and female animals, and there was a positive significant correlation between liver coefficient and the drug doses (Figure 4). Kidney coefficient of male animals were increased significantly by three doses of AGO and GW117 (*p* < 0.01), in female animals, kidney coefficient were also increased in all drug-administered groups, but only three groups showed significant differences, AGO 400 mg/kg, GW117 800 mg/kg, and GW117 200 mg/kg (*p* < 0.05).

**Table 3:** Effects of AGO and GW117 on organ weight and organ coefficients in rats (*n* = 6).

Group	Dose (mg/kg)	Gender	Brain		Heart		Liver		Spleen		Lung		Kidney		Testes/ovary	
									
			Weight (g)	Coefficient (g/100 g)	Weight (g)	Coefficient (g/100 g)	Weight (g)	Coefficient (g/100 g)	Weight (g)	Coefficient (g/100 g)	Weight (g)	Coefficient (g/100 g)	Weight (g)	Coefficient (g/100 g)	Weight (g)	Coefficient (g/100 g)
Vehicle	0.5% CMC	Male	2.03 ± 0.05	0.56 ± 0.03	1.12 ± 0.03	0.31 ± 0.03	9.15 ± 0.03	2.53 ± 0.14	0.68 ± 0.14	0.19 ± 0.02	1.31 ± 0.12	0.36 ± 0.03	2.45 ± 0.18	0.68 ± 0.051	3.16 ± 0.36	0.19 ± 0.02
AGO	800		1.93 ± 0.06	0.61 ± 0.05	1.01 ± 0.05	0.32 ± 0.05	11.8 ± 0.05	3.70 ± 0.17^∗∗^	0.65 ± 0.17	0.20 ± 0.02	1.29 ± 0.14	0.41 ± 0.04	2.67 ± 0.23	0.84 ± 0.06^∗∗^	3.03 ± 0.22	0.20 ± 0.02
	400		1.92 ± 0.12	0.56 ± 0.03	1.16 ± 0.03	0.34 ± 0.03	11.370.037	3.33 ± 0.03^∗∗^	0.69 ± 0.03	0.20 ± 0.04	1.41 ± 0.34	0.41 ± 0.01	2.93 ± 0.16	0.87 ± 0.10^∗∗^	3.18 ± 0.23	0.20 ± 0.04
	200		1.96 ± 0.07	0.59 ± 0.03	1.12 ± 0.03	0.34 ± 0.03	10.090.037	3.03 ± 0.21^∗∗^	0.69 ± 0.21	0.21 ± 0.02	1.48 ± 0.22	0.45 ± 0.04	2.58 ± 0.2	0.78 ± 0.04^∗^	3.19 ± 0.4	0.21 ± 0.02
GW117	800		1.93 ± 0.09	0.60 ± 0.03	1.08 ± 0.03	0.34 ± 0.03	11.320.039	3.55 ± 0.24^∗∗^	0.68 ± 0.24	0.21 ± 0.03	1.28 ± 0.19	0.4 ± 0.07	2.69 ± 0.37	0.84 ± 0.12^∗∗^	3.17 ± 0.11	0.21 ± 0.03
	400		1.87 ± 0.12^∗∗^	0.62 ± 0.08^∗^	1.06 ± 0.08	0.35 ± 0.08	10.020.083	3.32 ± 0.14^∗∗^	0.63 ± 0.14	0.21 ± 0.02	1.23 ± 0.14	0.41 ± 0.05	2.44 ± 0.24	0.81 ± 0.07^∗∗^	3.02 ± 0.34	0.21 ± 0.02
	200		1.92 ± 0.05	0.60 ± 0.02	1.11 ± 0.02	0.35 ± 0.02	9.44 ± 0.02	2.97 ± 0.20^∗∗^	0.69 ± 0.20	0.22 ± 0.03	1.41 ± 0.23	0.44 ± 0.07	2.72 ± 0.2	0.85 ± 0.03^∗∗^	3.30 ± 0.31	0.22 ± 0.03
Vehicle	0.5% CMC	Female	1.83 ± 0.11	0.78 ± 0.03	0.83 ± 0.03	0.35 ± 0.02	6.72 ± 0.02	2.88 ± 0.08	0.53 ± 0.08	0.23 ± 0.02	1.1 ± 0.17	0.47 ± 0.06	1.77 ± 0.15	0.76 ± 0.05	0.17 ± 0.03	0.23 ± 0.02
AGO	800		1.78 ± 0.15	0.85 ± 0.09	0.71 ± 0.09	0.34 ± 0.02	9.85 ± 0.02	4.67 ± 0.28^∗∗^	0.46 ± 0.28	0.22 ± 0.03	0.96 ± 0.04	0.46 ± 0.02	1.74 ± 0.11	0.83 ± 0.07	0.14 ± 0.02	0.22 ± 0.03
	400		1.79 ± 0.09	0.80 ± 0.07	0.80 ± 0.03	0.36 ± 0.03	8.73 ± 0.03	3.89 ± 0.14^∗∗^	0.43 ± 0.14	0.19 ± 0.02	1.36 ± 0.65	0.60 ± 0.26	1.89 ± 0.05	0.84 ± 0.05^∗^	0.19 ± 0.05	0.19 ± 0.02
	200		1.86 ± 0.05	0.83 ± 0.04	0.66 ± 0.04	0.30 ± 0.07	8.12 ± 0.07	3.62 ± 0.12^∗∗^	0.49 ± 0.12	0.22 ± 0.04	1.11 ± 0.09	0.49 ± 0.02	1.86 ± 0.16	0.83 ± 0.04	0.19 ± 0.04	0.22 ± 0.04
GW117	800		1.76 ± 0.04	0.82 ± 0.05	0.78 ± 0.05	0.37 ± 0.03	8.57 ± 0.03	4.00 ± 0.16^∗∗^	0.43 ± 0.16	0.20 ± 0.06	1.02 ± 0.15	0.47 ± 0.05	1.86 ± 0.23	0.86 ± 0.07^∗^	0.15 ± 0.02	0.20 ± 0.06
	400		1.81 ± 0.06	0.87 ± 0.05	0.73 ± 0.05	0.35 ± 0.02	7.59 ± 0.62	3.65 ± 0.39^∗∗^	0.43 ± 0.39	0.20 ± 0.03	1.05 ± 0.08	0.50 ± 0.03	1.73 ± 0.14	0.83 ± 0.04	0.14 ± 0.05	0.20 ± 0.03
	200		1.83 ± 0.05	0.85 ± 0.06	0.78 ± 0.06	0.36 ± 0.03	7.71 ± 0.03	3.57 ± 0.35^∗∗^	0.52 ± 0.35	0.24 ± 0.02	1.3 ± 0.24	0.60 ± 0.14	1.86 ± 0.26	0.86 ± 0.11^∗^	0.13 ± 0.04	0.24 ± 0.02


*Vs. 0.5% CMC, ^∗^*p* < 0.05, ^∗∗^*p* < 0.01.*

### Effects of Ago and GW117 on Pathological Examination

Except for the liver and kidneys, pathological damage was not observed in organs of the brain, heart, spleen, lungs, and testes/ovary (data not shown).

**FIGURE 5:**
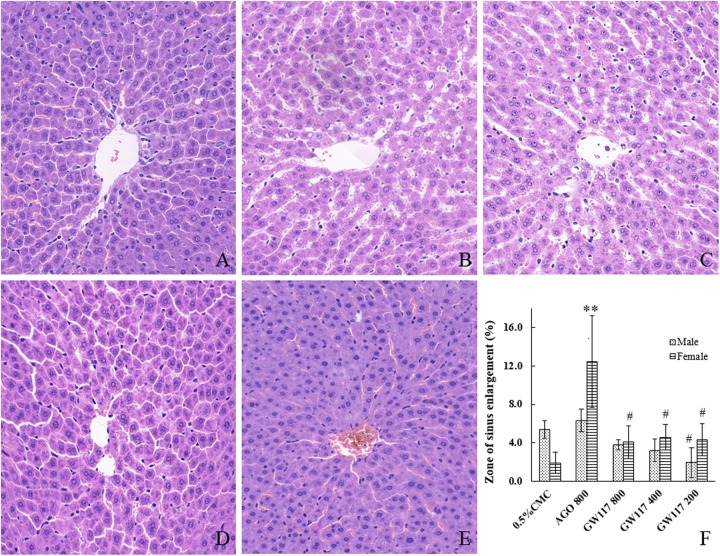
Effects of AGO and GW117 on the histopathological changes of the liver in rats. **(A)** 0.5% CMC; **(B)** AGO 800 mg/kg; **(C)** GW117 800 mg/kg; **(D)** GW117 400 mg/kg; **(E)** GW117 200 mg/kg; **(F)** zone of liver sinus enlargement vs. 0.5% CMC group, ^∗^*p* < 0.05, ^∗∗^*p* < 0.01; vs. the AGO group, ^#^*p* < 0.05 (*n* = 6).

The normal liver cells were polygonal (Figure 5A), and had abundant cytoplasm, One or two large and round nuclei with a smooth nuclear membrane. Some of the cells had a small number of lipid droplets. All cells were arranged radially around the central vein to form the hepatic sinusoids. There were scattered red blood cells and Kupffer cells in the hepatic sinusoids.

In AGO 800 mg/kg group, the hepatocytes were nearly polygonal with one or two nucleoli, but the nuclear membrane was not smooth (Figure 5B). There were many lipid droplets in the cytoplasm of cells, especially those around the central vein. The nucleus of a small number of hepatocytes disappeared. The hepatic sinusoids near the central vein were significantly dilated, and these changes were more pronounced in the central region of the liver lobe; hepatic cords arranged slightly disordered. Many red blood cells and Kupffer cells were scattered in hepatic sinusoids.

The morphology of liver cells of GW117 800 mg/kg group was near normal (Figure 5C), the number of cells deposited with fat droplets were less than that of AGO 800 mg/kg group, the hepatic cords are arranged regularly, and the area of hepatic sinusoidal dilatation is smaller than that of AGO group (Figure 5F). There were many red blood cells and Kupffer cells scattered in the hepatic sinusoids.

In GW117 400 and 200 mg/kg groups, the number of cells with fatty degeneration cells decreased and the area of hepatic sinusoidal dilatation reduced, the liver tissue structure of the 200 mg/kg group is close to normal (Figure 5D–F).

**FIGURE 6:**
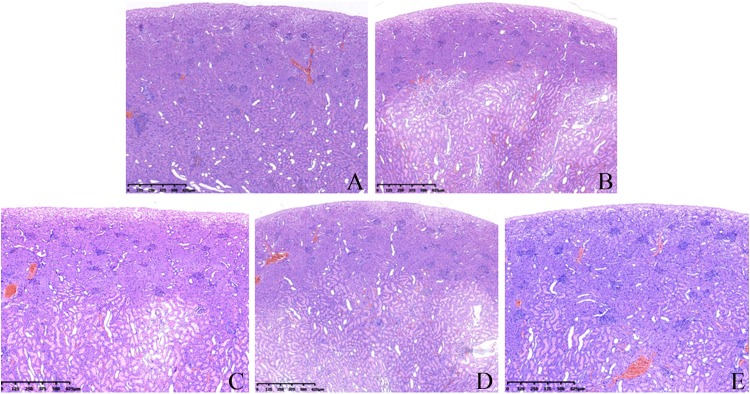
Effects of AGO and GW117 on the histopathological changes in the kidneys in rats. **(A)** 0.5% CMC; **(B)** AGO 800 mg/kg; **(C)** GW117 800 mg/kg; **(D)** GW117 400 mg/kg; **(E)** GW117 200 mg/kg.

The kidneys of the control group showed a normal structure (Figure 6A), and the dividing line between the cortex and medulla was clear, the cortical glomeruli were round or nearly round, and a small number of erythrocytes could be seen in glomerular capillaries. The renal tubular structure was clear, and the cells were arranged neatly.

In the AGO 800 mg/kg group (Figure 6B), the dividing line between the renal cortex and medulla was unclear, and the cortical layer was clearly thinned. A wide range of cortical and medullary renal tubules were dilated markedly, and the shape of some glomeruli were irregular, while a small number of inflammatory cells infiltrated in the tubular space or near the glomeruli.

The histopathological changes in the kidneys of the GW117 800 mg/kg group were similar to those of the AGO 800 mg/kg group (Figure 6C). The cortical layer was clearly thinner than the control group, but thicker than the AGO 800 mg/kg group, the renal tubules in the cortical and medullary areas were obviously dilated too and the structure of glomeruli were clear, while inflammatory cells infiltrated outside of some glomeruli.

In the GW117 400 mg/kg group (Figure 6D), the medullary boundary was clear, and area of renal tubule dilatation was reduced. The glomeruli shape and size were near normal. In the GW117 200 mg/kg group (Figure 6E), the dividing line of the renal cortex and medulla was clear, and the thickness of the cortex was close to normal. The area of renal tubule dilatation was obviously reduced, and the glomeruli morphology were regular.

## Discussion

In recent years, the incidence of depression has gradually increased according to WHO (World Health Organization), and there are about 350 million people that suffer from depression around the world, covering all age groups. It is expected to become the second largest disease in the world by 2020 (Dhir, 2017). Therefore, it is imperative to develop antidepressants with good efficacy and low adverse reactions. In the present study, we explored the sub-acute oral toxic action of GW117, a new derivative of AGO, and it compared to AGO in order to evaluate its development potential.

Throughout the experiment, AGO and GW117 had no negative effects on weight gain and the general state of the animals. The hematological results showed that the most obvious effect of these two drugs was a reduction of the platelet count in male animals, but not in female animals.

As reported in the literature, AGO can cause liver damage (de Gage et al., 2018), and GW117 as a derivative of AGO could cause liver injury too. In this study, liver injury indicators such as ALT, AST, ALP and total bilirubin were detected. AGO high and medium doses could increase ALT, total bilirubin in male animals and GW117 had no effect on the four indicators. AGO increased ALT, ALP and total bilirubin in female animals, while GW117 increased ALP only. Serum level augment of ALT and AST occurred due to the enzyme leakage from the liver into the circulation as a consequence of hepatocyte damage (Hussain et al., 2012). AGO caused a significant increase in ALT levels in all genders, indicating that AGO could cause hepatocyte rupture damage, while GW117 did not. Total bilirubin and ALP levels are indicators of hepatobiliary injury, AGO could significantly increase total bilirubin in males, and increase ALP and total bilirubin in females, indicated hepatobiliary injury in both genders, and more noticeably in females. GW117 had no effect on ALP and total bilirubin levels in male animals, but increased ALP in female animals, so GW117 did not show hepatobiliary injury in males, but in females. This results suggests that AGO causes more obvious hepatobiliary injury than GW117, and that female animals are more susceptible to this kind of injury. In brief, AGO could cause mild to moderate hepatocyte and hepatobiliary injury in all genders, while only mild hepatobiliary injury was caused by GW117 in females.

Agomelatine and GW117 had no negative effects on total plasma protein, albumin, prealbumin, TG and CHO, suggest that they did not caused significant damage to liver synthesis function during the test period.

The results of blood uric acid, creatinine and urea nitrogen showed that for male animals, AGO and GW117 can increase urea nitrogen. For female animals, AGO can significantly increase creatinine and urea nitrogen, GW117 can only increase urea nitrogen. This result suggests that the damaging effects of AGO on renal function is more pronounced than that of GW117.

Organ weight and organ coefficient results suggested that liver and kidneys are the main organs affected by AGO and GW117. Three doses of AGO and GW117 could significantly increase liver coefficient and kidney coefficient in all genders, suggesting that these two drugs have the potential to cause toxicity in the liver and kidney. The Pearson correlation analysis showed that the increase in liver coefficient caused by AGO and GW117 was positively correlated with the dose administered.

It was reported that the AGO-caused hepatic enzyme induction was more pronounced in rats, as a consequence of induction, and the animals showed enlarged livers and/or hepatocellular hypertrophy (European Medicines Agency, 2009). This phenomenon can partly explain the cause of liver enlargement, but liver pathological damage may be a more important reason. To further explore their relationship, a liver histopathological examination was performed. When compared to the normal liver tissue in the control group, AGO could cause different types of pathological changes, including hepatocyte damage, steatosis and punctate necrosis, etc. The most important tissue damage was the hepatic sinusoids expansion in the central region of the liver lobe, and the hepatocytes arrangement was disordered. Figure 5F shows the statistical results of the area of the hepatic sinusoidal expansion. The area of hepatic sinusoidal dilatation caused by the AGO 800 mg/kg was significantly larger than that of the control group in female rats (*p* < 0.01), while there was no statistical difference in male rats. The areas of hepatic sinusoidal dilation caused by the three doses of GW117 were significantly reduced compared to that of the AGO at 800 mg/kg in female animals (*p* < 0.05). The extent of hepatic sinus dilation was more obvious in females than in males, consistent with the results of ALP and total bilirubin.

The histopathological examination of the kidneys showed that both AGO and GW117 had mild renal damage. The most common manifestations are the expansion of the renal tubules in the inner cortex and the medulla, disordered arrangement of renal tubular cells, loss of the nucleus of some renal tubular cells, and the fall of the villus layer of the renal tubule. These pathological changes lead to a cortical thinning, and the dividing line between the cortex and the medulla was unclear. The most obvious pathological changes in kidneys were caused by the AGO at 800 mg/kg, while kidney injury caused by the GW117 at 800 mg/kg was equivalent to that of the AGO 800 mg/kg. When the dose of GW117 decreased, the area of tubular dilatation decreased, and the thickness of the cortex increased gradually. There was inflammatory cell infiltration outside a small number of glomeruli, and interstitial small blood vessels were slightly congested. There was no sex-difference in renal histopathological changes caused by AGO and GW117. There are few reports on kidney damage caused by AGO, and the report of the European Medicines Agency stated that no biologically relevant effects were observed in renal function. Instead, some articles have reported that AGO has a kidney protective effect, and it is believed that antioxidant and secretion inhibition of pro-inflammatory cytokines by AGO is responsible for the improvement in the kidneys (Başol et al., 2016; Demirdaş et al., 2016; Karaman et al., 2016; Rossetti et al., 2018). This report is not consistent with the results of this study, possibly due to the high dose used and further studies are needed to clarify the characteristics of AGO’s nephrotoxicity. This will help to better understand the properties of kidney damage caused by GW117.

This novel compound was explored for the first time in a sex-dependent manner, the study obtained useful data to further explore the toxic effects of GW117 and to further develop it as an antidepressant. While rodents were used in this study, they do have species differences with people, and the number of animals were limited, the toxic reactions with low incidence were difficult to detect, and so the predictability of human toxicity was limited. It is therefore necessary to expand the number and increase the species of animals to obtain more effective and comprehensive data.

## Conclusion

The results of this sub-acute oral toxicity study indicates that GW117 can cause lighter tissue damage compared to AGO and has certain gender differences in some indicators. For male animals, the relatively obvious effects of GW117 were mild platelet count reduction, liver and renal functional and histopathological damage, while in female animals, GW117 did not cause thrombocytopenia, but also caused liver and renal function damage as well as pathological damage. GW117 caused much less serious liver damage than AGO, and females were more susceptible to hepatobiliary injury. These adverse effects were observed at levels high above the clinically designed doses (80, 160, and 32 times). As a derivative of AGO, GW117 was designed to be clinically administered at a dose of 5 mg/kg/day, and in this study high doses were administered to provide a full presentation of the possible adverse effects. At such high doses, GW117 only caused mild liver and kidney damage in all genders, mild platelets reduction in male animals, while the degree of damage caused was less severe than that caused by AGO. It can therefore be speculated that the adverse reactions caused by GW117 will be much lower at clinical doses. GW117, with lower toxic effects, therefore has good potential to be further development as an anti-depressant agent.

## Data Availability

All datasets generated for this study are included in the manuscript and/or the supplementary files.

## Author Contributions

XL, ZJ, and WG devised and guided the research project. QY, JL, YM, LL, PX, JX, YL, and YC collected the original data. XL, WG, XZ, and MX analyzed the results. XL, XZ, and QY wrote and revised the manuscript.

## Conflict of Interest Statement

WG was employed by company Beijing Guangwei Pharmaceutical Technology Co., Ltd. The remaining authors declare that the research was conducted in the absence of any commercial or financial relationships that could be construed as a potential conflict of interest.
